# The roles and mechanisms of senescence-associated secretory phenotype (SASP): can it be controlled by senolysis?

**DOI:** 10.1186/s41232-022-00197-8

**Published:** 2022-04-02

**Authors:** Naoko Ohtani

**Affiliations:** Department of Pathophysiology, Graduate School of Medicine, Osaka City University, 1-4-3, Abeno-ku, Osaka, Japan

**Keywords:** Cellular senescence, Senescence-associated secretory phenotype, cGAS-STING pathway, Toll-like receptor, Tumor microenvironment, Senolysis

## Abstract

Cellular senescence is a state of irreversible cell cycle arrest that can be induced by a variety of potentially oncogenic stimuli, including DNA damage. Hence, senescence has long been considered to suppress tumorigenesis, acting as a guardian of homeostasis. However, recent studies have revealed that senescent cells exhibit the secretion of a series of inflammatory cytokines, chemokines, growth factors, and matrix remodeling factors that alter the local tissue environment and contribute to chronic inflammation and cancer. This senescence phenotype is termed as senescence-associated secretory phenotype (SASP) and is observed not only in cultured cells in vitro but also in vivo*.* Recently, the physiological and pathological roles of SASP have been increasingly clarified. Notably, several studies have reported that the intrinsic mechanism of SASP factor production is predominantly mediated through the activation of the cGAS-STING (cyclic GMP-AMP synthase-stimulator of interferon genes) pathway by aberrantly accumulated DNA fragments from the nucleus of senescent cells. In contrast, various extrinsic triggers of SASP also exist in vivo, for example, the SASP induction in hepatic stellate cells in the tumor microenvironment of obesity-associated liver cancer by the translocated gut microbial metabolites. Recently, the strategy for the elimination of senescent cells (senolysis) has attracted increasing attention. Thus, the role of SASP and the effects and outcomes of senolysis in vivo will be also discussed in this review.

## What is cellular senescence?

Cellular senescence is a state of permanent cell proliferation arrest induced by persistent DNA damage and other stress-induced signals. Cellular senescence was originally discovered as a proliferation limit observed in normal somatic cells after repetitive passage in culture and was termed as “replicative senescence” [1]. However, cellular senescence has since been reported not only in cultured cells but also in vivo in cells in various organisms, spanning from yeast to mammals [2]. Cellular senescence in vivo is caused by DNA damage-associated stress-induced senescence. Examples of DNA damage occurred in vivo include oxidative stress and exposure to UV irradiation or DNA damaging reagents. Recently, the role and mechanisms of the senescence-related phenotype and senescence-associated secretory phenotype (SASP) have been increasingly recognized as they are proposed to be associated with a variety of diseases [3–5]. In this review, the roles and mechanisms of SASP and the effect of eliminating senescent cells, called senolysis, have been summarized.

## Induction mechanism of cellular senescence

Cyclin-dependent kinase inhibitors (CDKIs), p16 and p21, are induced by persistent DNA damage; they play a role in inducing irreversible cell proliferation arrest, a phenotype that defines cellular senescence. DNA damage response signals initially stabilize p53 and induce p21 and CDKI. When DNA damage signals persist, p16 is induced through the Ets family transcription factor [6]. The two CDKIs, p21 and p16, collaborate to maintain the dephosphorylated form of RB protein, an essential cell cycle stopper, thereby contributing to strong irreversible cell cycle arrest [6]. Notably, p53 and p16 are inactivated in more than 50% of human cancers, illustrating that these senescence pathways are vital for suppressing the onset of cancer. Consistently, high expression of p16 and p21 is often used as senescence markers in vitro and in vivo.

Cellular senescence not only prevents the multiplication of cells harboring aberrant DNA that possibly causes tumorigenesis but also influences the tissue microenvironment through the development of a secretory phenotype. Cellular senescence is accompanied by a distinct secretory phenotype, SASP, which produces a variety of secreted proteins, cytokines, chemokines, growth factors, and proteases [7, 8]. Various roles and actions of SASP factors have been reported [9]. In an autocrine manner, SASP factors re-enforce cellular senescence of senescent cells themselves. SASP factors can also act in a paracrine manner, inducing senescence of surrounding cells, and this is termed as paracrine senescence [10]. The released chemokines from senescent cells as SASP factors reportedly act on immune cells, such as NK cells, and macrophages that can scavenge senescent cells [11]. Recently, it has become apparent that senescent cells transiently emerge during organ development in mammals, where SASP factors contribute to inducing the differentiation of surrounding cells and removal of unnecessary cells during development. Thus, SASP factors are also capable of cell-fate reprogramming [10–15].

Another important physiological role of SASP is repairing damaged tissues. Campisi et al. reported the transient emergence of senescent cells with SASP in subcutaneous fibroblasts, where the SASP played a role in tissue repair of damaged skin [12]. SASP factors from fibroblasts in damaged tissues recruit immune cells that contribute to the removal of damaged tissues. Simultaneously, senescent fibroblasts produce growth factors as SASP factors and promote the proliferation of skin progenitor cells to generate new skin. Senescent fibroblasts are eventually cleared by immune cells recruited from the new skin. Another example is liver injury, wherein hepatic stellate cells undergo cellular senescence to produce SASP factors and recruit immune cells. Immune cells play a role in eliminating senescent hepatic stellate cells (HSCs) to suppress excess collagen production and thereby prevent fibrosis [16]. Thus, repair of damaged tissue can be considered as a physiological role of SASP factors in vivo.

**Fig. 1:**
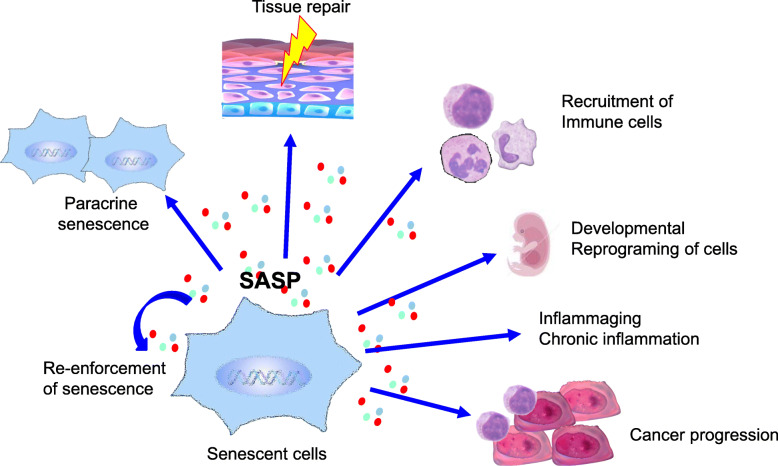
The effect of SASP factors. SASP factors can re-enforce cellular senescence in a autocrine manner. SASP factors can also act in a paracrine manner, inducing senescence of surrounding cells (paracrine senescence). SASP factors facilitate tissue repair and recruitment of immune cells. SASP factors are involved in development (developmental senescence). On the other hand, SASP factors are associated with chronic inflammation and cancer progression when persist

Apart from the functions described above, deleterious effects of SASP factors such as aging-associated inflammation and cancer have been suggested [17–19]. Indeed, several studies have reported that cancer-associated fibroblasts (CAFs) exhibit SASP [16–18, 20–23]. We have previously identified that hepatic stellate cells in the obesity-associated liver tumor microenvironment undergo senescence and exhibit tumor-promoting SASP factor production [16]. Since the pathological SASP tends not to be transient but to persist, thereby inducing undesirable outcomes such as cancer progression or chronic inflammation, clarifying the mechanism of SASP persistence is essential for controlling SASP (Fig. 1).

## Intrinsic factors for SASP induction: innate immunity through cGAS-STING pathway

As senescent cells that undergo SASP produce a variety of cytokines, chemokines, proteases, and growth factors, it is important to understand how these varieties are created. The most important cause of cellular senescence is persistent DNA damage. Hara et al. reported that persistent DNA damage strongly downregulates the expression of the histone dimethylating enzyme G9a, rendering the genome to exhibit more open chromatin to induce SASP factor gene expression [24]. Furthermore, the same group reported that DNA damage response in senescent cells is accelerated in the late stage of cellular senescence, producing small DNA fragments by cytokinesis block with proceeded nuclear division [25–27]. These abnormal cytoplasmic DNA fragments are supposed to trigger DNA sensors and exert innate immune inflammatory responses.

Recently, several reports have demonstrated that abnormal cytoplasmic DNA fragments produced during cellular senescence act as a ligand of the DNA sensor, cGAS-STING, and provoke a series of cytokine-producing pathways [26, 28–31]. Originally discovered as an innate immune receptor, cGAS recognizes DNA derived from cell-invading pathogens such as viruses and bacteria [32]. Interestingly, cGAS triggers the reaction to produce cyclic di-nucleotide, cyclic GMP-AMP, that is recognized by STING, thereby facilitating the type 1 interferon-producing pathway. Several studies have reported detailed mechanisms by which senescence-associated accumulation of cytoplasmic DNA fragments triggers the cGAS-STING pathway as follows. Persistent cellular senescence reduces the expression of Lamin B1, located on the inner surface of the nuclear membrane [33]. The reduction of Lamin B1 protein destabilizes the nuclear structure, thereby creating micronuclei by chromatin extrusion from the nucleus. These micronuclei, in turn, trigger the cGAS-STING pathway to activate type 1 interferon production [28, 29, 31].

In addition, long interspersed element-1 (LINE-1 or L1) cDNA, a reverse transcription product from a retrotransposon LINE-1, also accumulates in senescent cells and triggers SASP [34]. In the mammalian genome, a repetitive DNA sequence called transposable elements (transposons and retrotransposons) is capable of moving and transposing the genome. In particular, the retrotransposon LINE-1 exhibits high mobilization activity. Notably, increased cytoplasmic cDNA fragments that were reverse-transcribed from LINE-1 retrotransposons were highly accumulated in senescent cells and triggered cGAS-STING pathway activation [34]. Moreover, the accumulated cytoplasmic cDNAs produced from LINE-1 retrotransposons have also been linked to aging-associated chronic inflammation [34]. Treatment with reverse-transcriptase inhibitors alleviated chronic inflammation, and this may hold potential as molecular targeting therapeutics for aging-associated chronic inflammation [34].

Gorbunova et al. showed that LINE-1 retrotransposon elements are de-repressed in SIRT6-deficient mice that exhibit accelerated aging. Cytoplasmic accumulation of LINE-1 cDNA in SIRT6-deficient mice triggered the cGAS-STING pathway to induce a type I interferon response, resulting in pathological inflammation. Inhibiting LINE-1 replication also significantly improved chronic inflammation in this context [35]. All these evidence indicates that the intrinsic trigger of SASP induction is associated with the abnormal accumulation of DNA fragments triggering the activation of the cGAS-STING pathway.

**Fig. 2:**
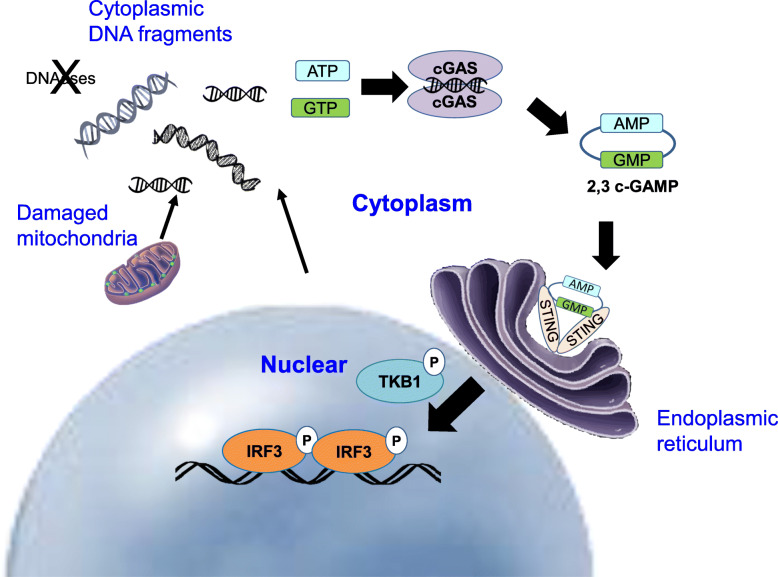
The intrinsic pathway of SASP induction in senescence: activation of cGAS-STING pathway by abnormal cytoplasmic DNA fragments. Abnormal cytoplasmic DNA fragments produced during cellular senescence act as a ligand of the DNA sensor, cGAS-STING. The cGAS, cyclic GMP-AMP synthase, triggers the reaction to produce cyclic di-nucleotide, cyclic GMP-AMP (cGAMP), that is recognized by STING, thereby facilitating the type 1 interferon-producing pathway activated by phosphorylated IRF3. Normally, DNases such as DNase2 and TREX1 degrade cytoplasmic DNA fragments emanating from the nucleus or damaged mitochondria. In senescent cells, the expression of these DNases is downregulated, resulting in cytoplasmic accumulation of DNA fragments. The remaining DNA fragments aberrantly activate the cytoplasmic DNA sensor, cGAS-STING

However, the mechanism by which DNA fragments accumulate in the cytoplasm had not been elucidated yet. Normally, DNases such as DNase2 and TREX1 degrade cytoplasmic DNA fragments emanating from the nucleus. However, the expression of these DNases, regulated by E2F, is downregulated in senescent cells, resulting in cytoplasmic accumulation of nuclear DNA. The remaining DNA fragments aberrantly activate the cytoplasmic DNA sensor, cGAS-STING pathway, inducing SASP through the induction of type 1 interferons. Downregulation of DNase2 and TREX1 is also observed in HSCs in the obesity-associated liver tumor microenvironment in vivo, and the blockade of this pathway prevented SASP in HSCs and obesity-associated hepatocellular carcinoma development in mice [26]. Although the aberrant accumulation of senescence-associated DNA cytoplasmic fragments is not derived from pathogens such as viruses or bacteria, they provoke innate immune responses through the cGAS-STING DNA sensor, contributing to the onset of SASP. Thus, activation of the cGAS-STING pathway plays a pivotal role as an intrinsic pathway for SASP induction (Fig. 2) [26].

## Role of SASP in tumor microenvironment

**Fig. 3:**
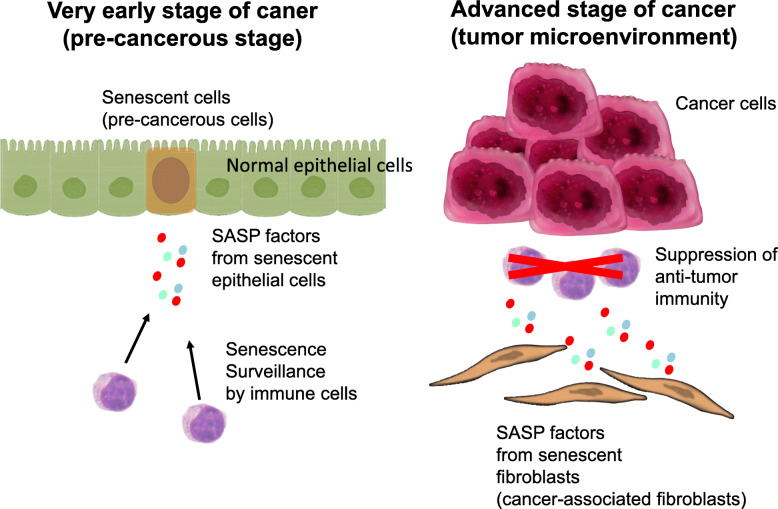
The role of SASP in tumor microenvironment. The effects of the SASP depends on the tumor stage. In precancerous cells (or in a very early stage of cancer), which are known to be in the senescent state, the effects of the SASP factors from the precancerous cells are predominantly tumor-suppressive, recruiting immune cells to exclude precancerous senescent cells (senescence surveillance). However, in advanced stage of tumor tissues, the SASP factors from senescent cancer-associated fibroblasts (CAFs) support the proliferation of cancer cells and promote tumor progression

When considering the role of SASP in tumor development, it should be considered the type of cells undergoing senescence and SASP, i.e., whether they are precancerous epithelial cells or stromal cells (e.g., CAFs in the tumor microenvironment). Additionally, it is important to determine whether the cancer is in an early stage or advanced stage, as SASP in precancerous epithelial cells plays a preventive role against tumorigenesis, while in advanced cancer, SASP in stromal fibroblasts promotes tumor progression (Fig. 3).

Cellular senescence was originally identified as an important tumor suppression mechanism, and cellular senescence is known to be detected precancerous cells [36]. Hence, the clearance of precancerous senescent cells can prevent the onset of cancer [37, 38]. Accordingly, the clearance system of senescent cells is called the senescence surveillance system. This system was first demonstrated in a liver cancer model [37, 38], wherein SASP factors from precancerous senescent hepatocytes recruited immune cells for the clearance of precancerous cells. Thus, rapid clearance of senescent cells and subsequent cancelation of SASP are important for suppressing early onset of cancer [39].

## Role of SASP in cancer progression

Stromal cells, particularly CAFs in the tumor microenvironment, play deleterious roles in tumor progression. We have previously shown that HSCs exhibit tumor-promoting SASP in the liver tumor microenvironment [16, 40]. Interestingly, high-fat diet-induced obesity increases gram-positive gut microbiota that produces secondary bile acid, deoxycholic acid (DCA) [16]. The enterohepatic circulation of DCA induces DNA-damage-induced cellular senescence in HSCs, with simultaneous SASP induction. We confirmed that mice lacking IL-1β an upstream regulator of SASP factor induction showed cellular senescence of HSCs with a strong reduction in the expression of SASP factors [16]. These mice also showed a decline in liver tumor formation, suggesting that IL-1β mediated pathway in HSCs plays a role in obesity-associated liver tumor progression. Moreover, depletion of HSCs by knocking down HSP47 expression in vivo significantly suppressed obesity-associated liver tumor formation [16]. These results suggest that senescent HSCs play a key role in obesity-associated liver cancer development through the secretion of SASP factors.

Other reports have also indicated that IL-6 produced from stromal cells in the tumor microenvironment of skin cancer activated myeloid-derived suppressor cells, suppressing anti-tumor immunity [41]. In clinical practice, therapy-induced senescence, a state of stable cell proliferation arrest induced by cancer treatments such as chemotherapy and radiation, can induce SASP [42]. Notably, the promotion of breast cancer metastasis and therapy resistance by therapy-induced senescence in stromal cells has been observed [43, 44].

## Extrinsic SASP induction in obesity-associated liver tumor microenvironment

As previously described, we had reported an increase in blood DCA levels by DCA producing gram-positive bacteria in neonatal 7, 12-dimethylbenz[a]anthracene (DMBA)-treated and high fat diet (HFD)-fed mice, that promoted obesity-associated liver cancer development [16]. However, liver tumors were not observed in normal diet-fed mice treated with neonatal DMBA [16, 40]. In order to elucidate the mechanism by which HFD-induced obesity promotes liver cancer, we focused on changes in the gut microbial profile. Consistent with previous reports, the gram-positive gut microbiota was greatly increased in HFD-fed mice. Therefore, we focused on the dynamics of lipoteichoic acid (LTA), a microbe-associated molecular pattern of gram-positive bacteria. When we performed liver carcinogenesis experiments in mice lacking TLR2 (Toll-like receptor 2), a receptor that recognizes LTA, TLR2-deficient mice developed significantly reduced liver tumors. Moreover, LTA accumulated in the livers of HFD-fed mice through a leaky gut, suggesting that HFD-associated liver cancer was promoted by the LTA-TLR2 pathway. Detailed analysis revealed that liver tumor formation was accelerated by the suppression of anti-tumor immunity by prostaglandin E_2_ (PGE_2_) overproduction, which was mediated by COX-2 induced by LTA from DCA-induced senescent HSCs. LTA also induces a variety of SASP factors. Thus, LTA derived from HFD-increased gram-positive gut microbiota plays a role as an extrinsic factor for SASP induction. Transfer of LTA to the liver also suggests that long-term HFD intake induces leaky gut formation. Together, the data indicates that PGE_2_ is crucial for suppressing anti-tumor immunity. We also noted that one of the receptors for PGE_2_, EP4, was strongly upregulated in the liver tumor region. Hence, we pre-treated mice with an EP4 antagonist. Pre-treatment with an EP4 antagonist significantly prevented obesity-associated liver tumor formation, accompanied by an increased number of CD69-positive activated CD8 T lymphocytes and decreased number of PD-1-positive suppressed CD8 T lymphocytes. COX-2 upregulation and PGE_2_ overproduction have been reported in human non-alcoholic steatohepatitis-associated liver tumors with less fibrosis and high lipid accumulation, indicating that these mechanisms may be conserved in certain types of human liver cancer [40].

## Senolysis

The accumulation of senescent cells in vivo exerts deleterious effects on SASP through inflammatory/tumor-promoting factor secretion. Hence, the development of new strategies to specifically eliminate senescent cells, termed “senolysis,” is anticipated. Recently, studies on senolysis have increasingly attracted attention, since healthy longevity has been successfully demonstrated in several genetically engineered mouse models after senolysis [19, 45, 46]. Accordingly, screening for senolysis drugs has been greatly promoted and some sets of senolytic drugs have been discovered [47, 48]. Dasatinib and quercetin was one of the first set of senolytic drugs [48, 49]. The combination of these two drugs led to decreased number of senescent cells in aged or irradiated mice [49]. However, the mechanism behind the induction of senescent cell death induced by these drugs remains unclear.

Hara et al. identified a BET family protein degrader (BETd) as a promising senolytic drug. BETd provokes senolysis through two independent but integrated pathways: the attenuation of non-homologous end joining (NHEJ) and the activation of the autophagic pathway. Senescent cells characteristically cease proliferating, and thus, only NHEJ functions as a DNA repair tool for double-strand breaks. Moreover, the autophagic pathway is downregulated in long-term senescent cells [50]. Therefore, autophagic activation by BETd induces autophagic cell death in senescent cells. Treatment with BETd also eliminates senescent HSCs in the tumor microenvironment in obesity-associated liver tumors in vivo, leading to a reduction in liver cancer development. These discoveries on the senolytic function of BETd unveiled a novel vulnerability in senescent cells [50]. More recently, Nakanishi et al. reported that the glutaminolysis pathway is accelerated in senescent cells and a glutaminolysis inhibitor, GSL1, induced senolysis and ameliorated various age-associated disorders [51]. Targeting therapy-induced senescent cells by senolysis has also been reported [52]. Cancer therapies using DNA-damaging reagents can trigger cellular senescence of tumor cells and surrounding cells, and SASP factors secreted from senescent cells may negatively affect the tumor microenvironment. Accordingly, eradication of therapy-induced senescent cells has been shown to improve the outcome of liver cancer therapy [52].

## Conclusions

As mentioned above, recent findings have revealed the vulnerabilities of senescent cells. Accordingly, studies have shown that elimination of senescent cells induces extension of a healthy life span and improvement of cancer. However, it has been suggested that elimination of senescent liver sinusoidal endothelial cells disrupts blood-tissue barriers and promotes perivascular liver fibrosis, and mice tend to die earlier [53]. These data suggest that senescent liver sinusoidal endothelial cells play important structural and functional roles in aging organisms [53]. As senescent cells may have a role in organ structure, more restricted use of senolysis should be considered, and more detailed mechanisms for senolysis should be elucidated. Thus, further studies will open up possibilities for the control of senescent cells and the beneficial use of senolysis to ameliorate senescence-associated diseases.

## Data Availability

Not applicable.

## Abbreviations

BETd: BET family protein degrader
CDKIs: Cyclin-dependent kinase inhibitors
CAFs: Cancer-associated fibroblasts
cGAS-STING: Cyclic GMP-AMP synthase-stimulator of interferon genes
DCA: Deoxycholic acid
DMBA: 7, 12-Dimethylbenz[a]anthracene
HFD: High fat diet
HSCs: Hepatic stellate cells
LINE-1: Long interspersed element-1
LTA: Lipoteichoic acid
NHEJ: Non-homologous end joining
PGE_2_: Prostaglandin E_2_
SASP: Senescence-associated secretory phenotype
TLR2: Toll-like receptor 2
